# A Slanted-Finger Interdigitated Transducer Microfluidic Device for Particles Sorting

**DOI:** 10.3390/mi16040483

**Published:** 2025-04-20

**Authors:** Baoguo Liu, Xiang Ren, Tao Xue, Qiang Zou

**Affiliations:** 1 School of Microelectronics, Tianjin University, Tianjin 300072, China; lbg_6227@tju.edu.cn (B.L.); xren@tju.edu.cn (X.R.); 2 State Key Laboratory of Advanced Materials for Intelligent Sensing, Tianjin University, Tianjin 300072, China; 3 Center of Analysis and Testing Facilities, Tianjin University, Tianjin 300072, China; xuetao@tju.edu.cn; 4 Tianjin International Joint Research Center for Internet of Things, Tianjin 300072, China; 5 Tianjin Key Laboratory of Imaging and Sensing Microelectronic Technology, Tianjin University, Tianjin 300072, China

**Keywords:** microfluidics, particle sorting, broadband SFIT, adjustable-TSAW

## Abstract

Sorting particles or cells of specific sizes in complex systems has long been a focus of many researchers. Acoustic surface waves, which generate acoustic radiation forces on particles or cells and, thus, influence their motion, are commonly used for the non-destructive separation of particles or cells of specific sizes. In previous studies, the frequency of acoustic surface wave generation has been limited by the interdigitated transducer (IDT). To extend the effective operating frequency range of the IDT, a slanted-finger interdigitated transducer (SFIT) with a wide acoustic path and multiple operating frequencies was designed. Compared with traditional acoustic sorting devices, which suffer from a limited frequency range and narrow acoustic paths, this new design greatly expands both the operating frequency range and acoustic path width, and enables adjustable operating frequencies, providing a solution for sorting particles or cells with uneven sizes in complex environments. The optimal resonance frequency is distributed within the 32–42 MHz range, and the operating frequencies within this range can generate a standing wave acoustic path of approximately 200 μm, thus enhancing the effectiveness of the operating frequencies. The microfluidic sorting device based on SFIT can efficiently and accurately sort polystyrene (PS) with particle sizes of 20 μm, 30 μm, and 50 μm from mixed PS microspheres (5, 10, 20 μm), (5, 10, 30 μm), and (5, 10, 50 μm), with a sorting efficiency and purity exceeding 96%. Additionally, the device is capable of sorting other types of mixed microspheres (5, 10, 20, 30, 50 μm). This new wide-acoustic-path, multi-frequency sorting device demonstrates the ability to sort particlesin a high-purity, label-free manner, offering a more alternative to traditional sorting methods.

## 1. Introduction

The classification of cells and particles plays a crucial role in medical diagnosis and therapy [1,2,3], chemical synthesis [4], environmental testing [5], and drug development [6]. With the advancement of related sciences and technologies, the range of techniques for cell and particle sorting has expanded, driving progress in fields such as medical testing [7], cancer diagnosis [8], and nanoparticle chemical synthesis [9]. In cancer diagnosis and treatment, the collection and detection of circulating tumor cells (CTC) are vital for diagnosing a patient’s condition [10]. The sorting of particles and cells using physical methods largely relies on the movement of particles and cells within microfluidic systems, which can be categorized into passive sorting [11,12] and active sorting (e.g., acoustic field [13], electric field [14], magnetic field [15]). Essentially, this process takes advantage of the differences in the inertial forces or the different net forces experienced by particles or cells in an external field, leading to distinct motion states that enable separation. Currently, in cell and particle sorting applications, flow cytometry and magnetic bead cytometry are well-established instruments, offering advantages such as high throughput and sensitivity. However, they also have drawbacks, including high equipment costs, the need for markers, and high operator skill requirements. To address these limitations and achieve low-cost, high-throughput, and high-efficiency sorting of particles and cells, many researchers have developed innovative solutions using microfluidic chips [16]. These innovative methods include the spiral approach, where a spiral structure is created on the microfluidic chip [17]. Chen et al. [18] used a femtosecond laser to engrave a spiral structure on glass and utilized the Dean force of the fluid within the spiral structure to sort the particles. This method has a simple design but is constrained by the size and density of the particles. Some scholars [19,20,21] have used the microcolumn method for sorting, where tiny column structures were designed in the microfluidic chip, guiding different microspheres through the chip for sorting. This method enables the large-scale sorting of cells and particles with a high screening accuracy. However, it requires a complex microfluidic channel design and carries the risk of particle clogging during use. Clark et al. [22] used the electrical properties of cells or particles to sort them by electrophoresis. Under the influence of the electric field, particles move at different speeds due to their varying charges, enabling sorting with a high resolution and accuracy. However, this method has notable limitations: it can only sort charged particles, it is restricted by the electrical properties of the particles, and it generates heat during the process, which can affect cell viability in cell sorting applications. The method of cell and particle sorting using surface acoustic waves (SAWs) [23] is characterized by non-contact, high throughput, and a high sorting efficiency. SAW is a weak acoustic wave generated by piezoelectric materials, which interacts with particles during propagation to shift them in the microfluidic chip, thereby achieving sorting. Compared to other methods, SAW can be adjusted based on particle size, density, and shape to achieve high-precision sorting, and it allows for direct screening without the need for pre-treatment or marker addition, offering high flexibility and adaptability. Currently, cell and particle sorting methods are increasingly inclined towards the combination of multiple approaches. The comprehensive utilization of the advantages of various methods has made sorting innovations more efficient and mature. Wu et al. [24] combined electric and acoustic fields, using the electric field for focusing and the acoustic field for sorting, which greatly improved sorting efficiency. Peng et al. [25] combined helical structures with acoustic fields, reducing the dependence of helical structure sorting on flow rate, thereby optimizing the sorting effect.

Sorting cells and particles in microfluidic chips using acoustic surface waves has been well developed in both theoretical and experimental research [26]. Acoustic surface waves can be broadly classified into two categories: standing surface waves (SSAWs) and traveling surface waves (TSAWs) [27]. SSAWs [13,28] are generated by the excitation of two opposing interdigital transducers (IDTs). SAWs transmitted between two IDTs will superimpose, creating regions of minimum and maximum acoustic pressure amplitudes, known as pressure nodes (PN) and wave antinodes, respectively. Cells or particles tend to aggregate towards the pressure nodes. Therefore, the successful separation of cells or particles depends on the effective control of the PNs’ position within the microfluidic channel. SSAWs can be employed in applications such as particle separation, cell manipulation [29], and droplet sorting [30], among others. TSAWs [31,32] are generated by the excitation of a single IDT and propagate perpendicularly to the direction of the IDT’s finger electrodes. Different excitation frequencies produce TSAWs with varying intensities, which exert acoustic radiation forces (ARFs) of different magnitudes on the particles. During their propagation, particles of different sizes experience different ARFs, allowing for their separation. TSAWs are also widely applied in various microfluidic manipulations. Mezzanzanica et al. [33] revealed the simulation-based mechanical principles underlying acoustic manipulation, providing a new mathematical model for the separation of particles of different sizes. Currently, there are many types of IDT designs, including a straight IDT (SIDT) [34], slanted-finger IDT (SFIT) [35,36], chirped IDT (CIDT) [37], and focused IDT (FIDT) [38,39], each with different parameter configurations and operating characteristics. Compared to the other three types of fork-finger transducers, the slant-finger IDT offers flexible and adjustable operating frequencies and acoustic path regions, along with a more sensitive acoustic amplitude. It is widely used to study the correlation between frequency and size, as well as the selective separation of particles of different sizes. The flexible and adjustable operating frequency of a SFIT used by Khan et al. [40] enabled the preparation of residue-free acoustic fluid particles, and Mutafopulos et al. [41] used a SFIT to achieve the selective encapsulation of cells and particles. Most of these studies focused on utilizing the flexible operating frequency of the SFIT, with less attention paid to the impact of separating cells and particles using multiple acoustic paths or to whether this affects cell viability.

**Figure 1:**
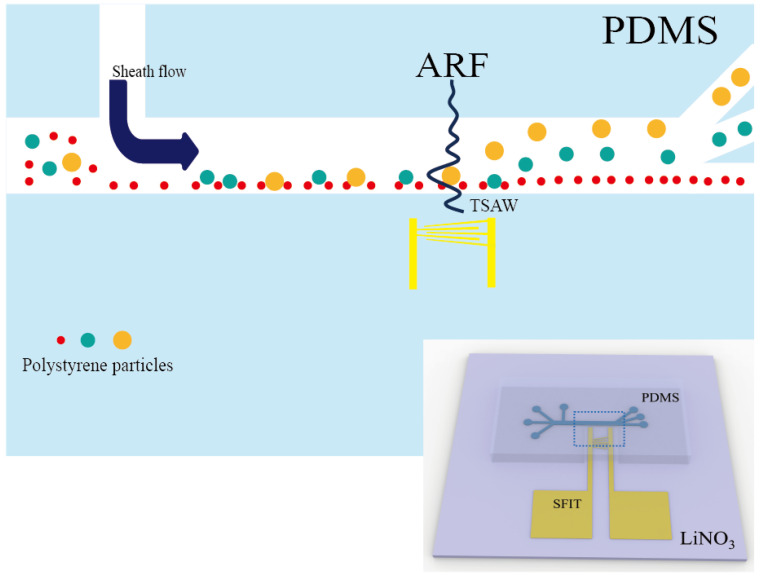
Conceptual diagram of device operation.

In this work, we designed a SFIT with a wide acoustic path and multiple operating frequencies, combined with our microfluidic chip featuring unidirectional sheath flow focusing. This system forms a high-efficiency sorting device with a wide frequency range and multi-size sorting capability (Figure 1). In the process of particle sorting using TSAWs, different particles exhibit different motion states under TSAWs with different frequencies. Therefore, TSAWs with a specific operating frequency can selectively sort particles of a particular size. In traditional acoustic surface wave research, researchers typically focus on sorting particles or cells of a single size, resulting in devices with a limited operating frequency range [24,25]. To address this issue, the design in this study uses a SFIT to excite TSAWs. The slanted, uniformly varying electrode pairs can be divided into multiple interconnected SIDTs, allowing for a frequency range rather than a single characteristic frequency. Within this range, different frequencies can be selected to sort particles of different sizes. In other words, within the operating frequency range, TSAWs at different frequencies can be generated in different areas, with each area sorting different types of particles. This approach increases the flexibility and robustness of the device. Additionally, the electrode length is increased, allowing for the tilt angle to vary within a ±5° range. Compared to narrow-band SFIT designs by other researchers [35,36] (with shorter electrode lengths and larger tilt angles), this design not only broadens the acoustic path width of the excited TSAWs but also ensures that the TSAWs are as perpendicular as possible to the direction of particle flow. This improves the stability of TSAWs’ operation at different frequencies and enhances the reliability of particle sorting. The electrode size of the SFIT is 30–50 μm, and its operating frequency ranges from 25 to 40 MHz. We increased the electrode length to 5000 μm, which expands the width of the acoustic path at different operating frequencies, making the TSAWs generated in different areas more uniform and maximally perpendicular to the direction of particle flow. This enhances the stability and accuracy of particle screening in different regions, allowing the device to be used for sorting particles or cells of various sizes. We validated the uniformity and usability of the wide acoustic path in our device design by using TSAWs excited by the SFIT at different frequencies to generate traveling wave acoustic paths in bare, mixed microsphere droplets. We observed the changes in the width, position, and verticality of the acoustic paths at various frequencies. Additionally, we verified the frequency tunability and flexibility of our device by sorting 20 μm, 30 μm, and 50 μm microspheres mixed with smaller-sized microspheres. In the particle sorting experiments, microspheres of 5 μm, 10 μm, 20 μm, 30 μm, and 50 μm were selected for separation. The 5 μm PS microspheres were used to mimic human erythrocytes, 10 μm PS for human leukocytes, 20 μm PS for circulating tumor cells in cancer patients, 30 μm PS for human macrophages or other microorganisms, and 50 μm PS for larger microorganisms. The broadness of the device’s operating frequency was verified by separating 20 μm, 30 μm, and 50 μm microspheres in combinations of 5 μm, 10 μm, 20 μm; 5 μm, 10 μm, 30 μm; and 5 μm, 10 μm, 50 μm. Based on this, 20 μm, 30 μm, and 50 μm microspheres were further separated simultaneously to validate the feasibility of separation in complex environments, thus laying the experimental foundation for further research on cell or microorganism separation. This novel multi-path frequency sorting device features an adjustable working frequency and a wide traveling wave acoustic path, enabling a flexible sorting capability. By adjusting different frequencies, particles of various sizes can be sorted during the particle sorting process. In actual cell sorting, the size of target cells is not fixed but varies within a certain size range. Therefore, this design is better suited to meet the practical requirements of cell sorting, providing a non-invasive and label-free method for cell collection. It can also be integrated with other functional units on the chip to enable more complex and diverse biomedical and clinical applications.

## 2. Theoretical Analysis

**Figure 2:**
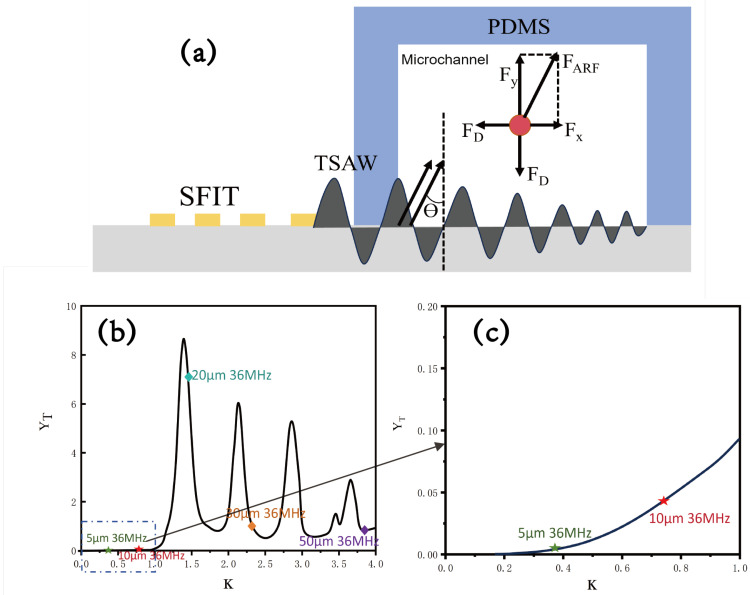
Force on particles: (**a**) force analysis of particles in the microfluidic channel within the acoustic field; (**b**) relationship between the acoustic radiation force coefficient $Y_{T}$ and κ; (**c**) variation in the acoustic radiation force coefficient for κ<1.

In the particle sorting process, the forces acting on the particles directly determine the sorting efficiency. In the acoustic field, particles and cells are primarily affected by gravity, buoyancy, Stokes resistance, and the acoustic radiation force. Among these, the forces that determine the lateral displacement of the particles are Stokes resistance and acoustic radiation force, with the acoustic radiation force (Figure 2a) playing a decisive role. By analyzing the acoustic radiation force acting on the particles, we can roughly assess whether the particles can be displaced and, thus, evaluate the feasibility of our device design.

Ideally, for a particle or cell suspended in an infinite stationary fluid medium with no net body force, perturbed by an acoustic wave of frequency *f*, assume that the fluid’s density, velocity, stress, and pressure are ρ, v, σ, and *P*, respectively. The particle or cell occupies a region ∂Ω(t) with a surface vector $\left(\vec{n}\right)$. The acoustic radiation force (ARF) acting on a particle or cell is obtained by surface integration of the fluid stress on the surface of the particle or cell. Since the instantaneous state of the acoustic radiation force cannot be resolved, we express the ARF using the averaged force over time [42,43]:

(1) $$F_{ARF}=<\oint_{\partial \Omega (t)}\sigma \cdot \vec{n}dS>$$

where the pointed brackets indicate that the solution is averaged over one oscillation period. In this equation, we account for the first-order viscous effects and the contribution of the acoustic flow. Based on the analysis of Jonas T. Karlsen [44], the equation is rewritten by integrating the force on the unit area of the particle and considering the inertial term of the particle as a correction during its motion:

(2) $$F_{ARF}=<\oint_{\partial \Omega 1}\left(\sigma -\rho \nu \nu \right)\vec{n}dS>$$

By applying perturbation theory to simplify the equations, a second-order expression for the acoustic radiation force can be derived:

(3) $$F_{ARF}=\oint_{\partial \Omega_{1}}\left[\sigma_{2}-\rho_{0}\langle \nu_{1}\nu_{1}\rangle \right]\vec{n}dS$$

The time-averaged tensor $\sigma_{2}$ over the oscillation period is given by

(4) $$<\sigma_{2}>=-\langle P_{2}\rangle I+\mu \left(\nabla \langle v_{2}\rangle +\nabla {\langle v_{2}\rangle }^{T}\right)$$

where I is the unit tensor. If second-order acoustic flow effects and the shear viscosity of the fluid are neglected, the acoustic radiation force can be further simplified as follows [45]:

(5) $$F_{ARF}=\oint_{\partial \Omega_{1}}\left[\left(\frac{1}{2\rho_{0}c_{0}^{2}}\langle {\left(\rho_{0}c_{0}^{2}\right)}^{2}\rangle -\frac{1}{2}\rho_{0}\langle \nu_{1}\cdot \nu_{1}\rangle \right)I-\rho_{0}\langle \nu_{1}v_{1}\rangle \right]\cdot \vec{n}dS$$

For spherical particles and cells, neglecting fluid viscosity and considering only elastic theory [46], the acoustic radiation force induced by exposure to the TSAW’s field can be expressed as follows:

(6) $$F_{ARF}=Y_{T}\cdot \bar{E}\cdot \left(\pi r^{2}\right)$$

where $Y_{T}$ is the acoustic radiation force coefficient of the TSAW, and ${\bar{E}}_{⊥}$ is the average energy density of the incident wave. For particles or cells of the same size under the influence of the same field strength, the acoustic radiation force is proportional to the coefficient $Y_{T}$. We define a dimensionless parameter κ ($\kappa =kR=\frac{2\pi fR}{c}$), where *f* is the excitation frequency of the acoustic surface wave, *R* is the radius of the particle or cell, and *c* is the velocity of the surface wave in the fluid. $Y_{T}$ can be expressed as a function of κ, and $Y_{T}$ is given by

(7) $$Y_{T}=-\frac{4}{k^{2}}\sum_{0}^{\infty }\left[\left(n+1\right)\left(P_{n}+P_{n+1}+2P_{n}P_{n+1}+2Q_{n}Q_{n+1}\right)\right]$$

$P_{n}$ and $Q_{n}$ can be expressed respectively as

(8) $$P_{n}=-\frac{{\left[F_{n}j_{n}\left(\kappa \right)-\kappa j_{n}^{\prime }\left(\kappa \right)\right]}^{2}}{{\left[F_{n}j_{n}\left(\kappa \right)-\kappa j_{n}^{\prime }\left(\kappa \right)\right]}^{2}+{\left[F_{n}y_{n}\left(\kappa \right)-\kappa y_{n}^{\prime }\left(\kappa \right)\right]}^{2}}$$

(9) $$Q_{n}=-\frac{\left[F_{n}j_{n}\left(\kappa \right)-\kappa j_{n}^{\prime }\left(\kappa \right)\right]\cdot \left[F_{n}y_{n}\left(\kappa \right)-\kappa y_{n}^{\prime }\left(\kappa \right)\right]}{{\left[F_{n}j_{n}\left(\kappa \right)-\kappa j_{n}^{\prime }\left(\kappa \right)\right]}^{2}+{\left[F_{n}y_{n}\left(\kappa \right)-\kappa y_{n}^{\prime }\left(\kappa \right)\right]}^{2}}$$

where $j_{n}$ and $y_{n}$ are the first and second kinds of Bessel functions, respectively, used to describe the propagation of acoustic waves in the particle or cellular region. The expression for $F_{n}$ can be given as follows:

(10) $$F_{n}=\frac{\kappa_{2}^{2}\cdot \rho_{f}}{2\rho_{p}}\cdot \frac{\frac{\kappa_{1}\cdot j_{n}^{\prime }\left(\kappa_{1}\right)}{\kappa_{1}\cdot j_{n}^{\prime }\left(\kappa_{1}\right)-j_{n}\left(\kappa_{1}\right)}-\frac{2n\left(n+1\right)j_{n}\left(\kappa_{2}\right)}{\left(n+2\right)\left(n-1\right)j_{n}\left(\kappa_{2}\right)+\kappa_{2}^{2}\cdot j_{n}^{\prime \prime }\left(\kappa_{2}\right)}}{\frac{\kappa_{1}^{2}\left[\sigma j_{n}\left(\kappa \right)/\left(1-2\sigma \right)-j_{n}^{\prime \prime }\left(\kappa_{1}\right)\right]}{\kappa_{1}\cdot j_{n}^{\prime }\left(\kappa_{1}\right)-j_{n}\left(\kappa_{1}\right)}+\frac{2n\left(n+1\right)[j_{n}\left(\kappa_{2}\right)-\kappa_{2}\cdot j_{n}^{\prime }\left(\kappa_{2}\right)]}{\left(n+2\right)\left(n-1\right)j_{n}\left(\kappa_{2}\right)+\kappa_{2}^{2}\cdot j_{n}^{\prime \prime }\left(\kappa_{2}\right)}}$$

where $\kappa_{1}$ and $\kappa_{2}$ are the values of κ for the corresponding wave speeds of longitudinal and transverse waves propagating inside the particles, respectively. σ is the Poisson’s ratio of the material, and $\rho_{f}$ and $\rho_{p}$ are the densities of the fluid and polystyrene microspheres, respectively. Based on the derivation of the expression for the function $Y_{T}$, we can plot the relationship between $Y_{T}$ and κ, providing the theoretical basis and working curve for screening different particles. Figure 2b,c demonstrate that when κ<1, $Y_{T}$ is very small, almost zero, and the particles are nearly unaffected by the acoustic radiation force. When κ>1, $Y_{T}$ exhibits a periodi variation with changes in κ, indicating that different particles have different $Y_{T}$ values and experience different acoustic radiation forces at the same frequency. This provides the basis for sorting. In mixtures of various particles, by adjusting the frequency, the $Y_{T}$ value corresponding to κ for one type of particle can be made much higher than that of other particles, enabling specific sorting of particles based on size. Based on the operating frequency of the device design, we can calculate the κ values for particles of different sizes at the response frequency to determine whether they can be separated. This serves as the basis for guiding the experiments. We label particles with different κ values at 36 MHz in Figure 2b,c. It is clearly observed that the particles experience different ARFs due to varying values of κ. By adjusting the frequency during movement, the κ values of the particles can be modified, enabling specific sorting of certain types of particles. We selected frequencies of 32 MHz, 34 MHz, and 36 MHz, as shown in Table 1, where the κ value changes with particle size. The corresponding $Y_{T}$ values are different in Figure 2b,c. With the adjustable operating frequency of the designed SFIT, this could be used as a reference for conducting the experiments.

**Figure 3:**
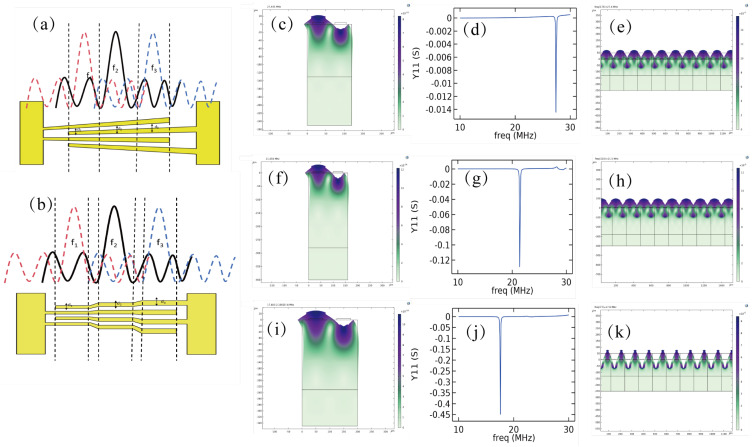
SFIT concept analysis and simulation: (**a**,**b**) SFIT operating mode analysis and simplification. (**c**–**e**) IDT operating frequency simulation with electrode width of 30 μm. (**f**–**h**) IDT operating frequency simulation with electrode width of 40 μm. (**i**–**k**) IDT operating frequency simulation with electrode width of 50 μm.

Since the operating frequency of a conventional IDT is determined at the design stage, in order to extend the device’s frequency range and enable it to adapt to particle or cell sorting requirements by adjusting the frequency, a slanted electrode finger pair, or SFIT, is chosen for the IDT design. This slanted finger pair behaves similarly to a conventional parallel finger pair and can be viewed as multiple parallel IDTs of different sizes connected together, offering a broader range of operating frequencies. These resonant frequencies can excite TSAWs at different frequencies, and by varying the device’s resonant frequency, particle or cell sorting can be achieved, thus reaching the desired sorting effect. The relationship model between SFIT and SIDT can be explained in detail in Figure 3. For slanted finger pairs of 30–50 μm, when the tilt angle and length are small enough, the electrodes can be roughly divided into link combinations of 30 μm, 40 μm, and 50 μm SIDTs. Simulations can be performed using simulation software to model and test the IDTs with these three finger widths. Under the same resonant waveform, a sequential change in resonant frequency can be observed, with the peak of the Y11 parameter shifting, demonstrating the wide range of the SFIT’s resonant frequencies. Based on the derivation of the above formula and model analysis, the theoretical foundation for the device design can be established. This is achieved by utilizing the wide resonance frequency range characteristics of the SFIT, combined with the acoustic radiation force factor $Y_{T}$ curves for particles or cells. By exploiting the different acoustic radiation forces experienced by particles at the same frequency, the resonance frequency can be adjusted to generate different acoustic paths, thus sorting particles and cells of different sizes.

## 3. Materials and Methods

### 3.1. Device Design and Fabrication

The device for acoustic surface wave markerless particle sorting, based on a slanted finger interdigitated transducer with a wide acoustic path and multiple operating frequencies, consists of a monolayer of polydimethylsiloxane (PDMS) and a piezoelectric substrate with a tapered SFIT. The SFIT is composed of Cr and Au (20 nm and 100 nm, respectively, with Cr acting as a transition layer metal to enhance the stability of the fork-finger electrode coating). It is fabricated by magnetron sputtering and stripping using a soft lithography mask on a 128°-Y cut X-propagation, double-polished LiNbO_3_ substrate. The electrode size of SFIT varies uniformly from 30 μm to 50 μm, and the size between each electrode also changes from 30 μm to 50 μm. The total aperture is 5000 μm, consisting of 20 finger pairs. Its resonant frequency can be estimated by dividing the speed of sound ($c_{s}$) in LiNbO_3_ by the wavelength (λ), i.e., $f_{\mathrm{resonant}}=\frac{c_{s}}{\lambda }$, and the operating frequency is roughly obtained to be in the range of 20–40 MHz. In the actual working process, the SFIT faces the microfluidic channel, which allows the TSAW to propagate along the direction perpendicular to the flow channel. The region of the SFIT retains an air chamber acoustic window favorable for TSAW propagation, reducing the attenuation of TSAW propagation in PDMS.

The microfluidic channel used in this experiment was fabricated by the inverted molding of microfluidic templates made from PDMS (SYLGARD 184 polymer/curing agent ratio of 10:1) through soft lithography on a wafer surface. It consists of four inlet channels (200 μm wide), three outlet channels (200 μm wide), and one main channel (400 μm wide, for sorting). The inlet section includes a polystyrene particle flow inlet channel and three sheath flow inlet channels. Two of the sheath flow inlet form a 30° angle with the sample inlet channel, and the last sheath flow inlet forms a 90° angle with the sample inlet channel. This arrangement ensures that the particles are focused on the side of the main channel close to the SFIT, which is beneficial for subsequent sorting. The angle between the three outlet channels is 22.5° for receiving the sorted particles. The fabricated PDMS is bonded to the LiNbO_3_ substrate after oxygen plasma treatment to fabricate the device.

**Figure 4:**
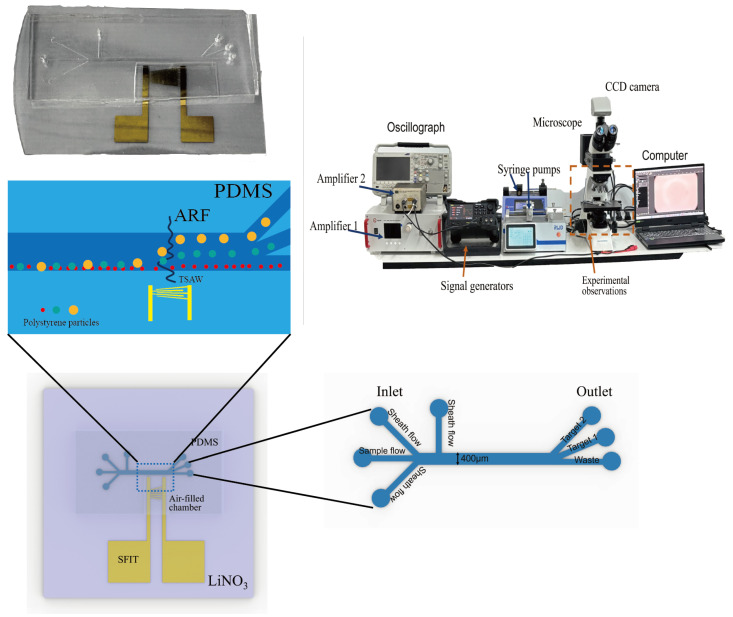
Device designconcepts with physical drawings and working platforms.

Figure 4 shows our working platform, the conceptual design of the device, and the physical image of the device. The assembled device was placed under a microscope (MIT1705097, CNOPTEC, Chongqing, China) for observation, which was equipped with a CCD camera (P60, AOR) with 19.8 megapixels to record the trajectory of particles inside the microfluidic channel. The PDMS tubing was connected to a dual-channel precision syringe pump (R462, RWD, Shenzhen, China) and a single-channel precision syringe pump (Pump11, Harvard Apparatus, Holliston, MA, USA) via tygon tubing (PTFE). The experiment began by generating a sample flow with a flow rate of 1 μL/min, two sheath flows at an angle of 30° with a flow rate of 1 μL/min, and one sheath flow at an angle of 90° with a flow rate of 5 μL/min. The resonant frequencies of the SFIT were measured using a vector network analyzer (ZNB8, ROHDE&SCHWARZ, Hong Kong, China) to determine the corresponding operating frequencies. The RF signal is generated by a function signal generator (DG2052, RIGOL, Suzhou, China), and the output voltage is amplified by an RF power amplifier (ATA-1200C, Aigtek, Xi’an, China) and a power amplifier (8447F, Hewlett-Packard, Palo Alto, CA, USA) to enhance the signal, improving the device’s performance.

### 3.2. Experimental Procedures

To test the SFIT designed in this experiment, polystyrene particles (25 mg/L, Tomicro) were used. The particle sizes of the samples tested were 5 μm, 10 μm, 20 μm, 30 μm, and 50 μm. Polystyrene particles of different sizes were mixed with a buffer solution consisting of deionized water and polyethylene glycol, with a mass fraction of 2% wt, to form the experimental test samples. The mixed samples tested include 5 μm, 10 μm, 20 μm; 5 μm, 10 μm, 30 μm; 5 μm, 10 μm, 50 μm; and 20 μm, 30 μm, 50 μm, mixed in a ratio of 10:5:1, 10:5:1, 10:5:1, and 1:1:1, respectively. In addition to using a vector network analyzer to validate the operating frequency of the device, the mixed microsphere droplets were exposed to test the SFIT, allowing for visualization of the standing wave generated by SFIT at different frequencies. In the particle sorting experiment, particles were injected into the microfluidic chip through a precision syringe pump, and the frequency and voltage values of the RF signal applied to the SFIT were adjusted to observe the sorting effect.

## 4. Results and Discussion

The device increases the selectivity of the operating frequency by using a slanted finger interdigitated transducer, which allows the resonant frequency of the device to vary over a range. The experimental results demonstrate the relationship between frequency and acoustic path, the effect of voltage flow rate on the sorting performance, the impact of different operating frequencies on particle separation, and the sorting performance for a variety of large-size mixed particles.

### 4.1. Operating Mode of SFIT

**Figure 5:**
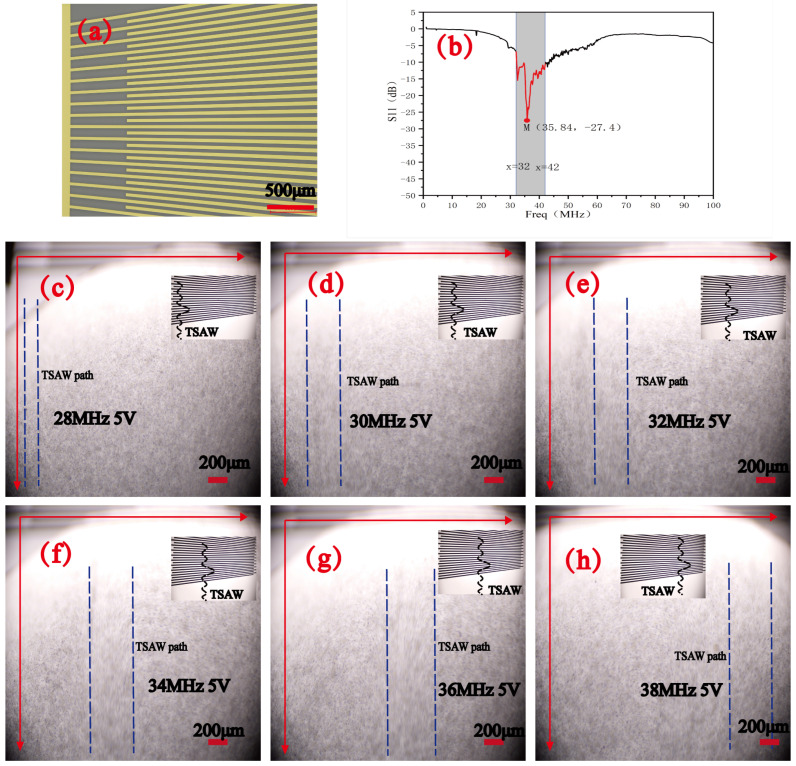
SFIT operating mode determination: (**a**) physical diagram of SFIT. (**b**) S11 parameters measured by a vector network analyzer. (**c**–**h**) Standing wave acoustic paths generated by the SFIT with a voltage of 5 Vpp, varying every 2 MHz in the 28–38 MHz range.

The physical diagram of the SFIT designed in this experiment is shown in Figure 5a. The SFIT features fork-finger electrodes that vary uniformly from 30 μm to 50 μm. The resonance frequency of the fork-finger electrodes was tested using a vector network analyzer in the frequency range of 9 kHz to 100 MHz, and the resonance frequencies obtained are shown in Figure 5b. The optimal operating frequency of the SFIT is in the range of 32 to 42 MHz. At 35.84 MHz, the return loss is minimized, and the energy conversion efficiency of the generated surface acoustic wave is highest at this frequency. Acoustic path experiments were conducted using this SFIT, with the frequency adjusted from 28 MHz to 38 MHz at 5 V, and measurements were taken at 2 MHz intervals. The results, shown in Figure 5c–h, and Video S1 demonstrate that as the frequency changes, the standing wave generated by the SFIT moves transversely and produces an acoustic path wider than 200 μm. As the frequency approaches 35.84 MHz, the acoustic path becomes wider, and the intensity of the surface acoustic wave increases. This experiment verifies the device’s characteristics of having a wide acoustic path and multiple operating frequencies, and it provides an experimental foundation for using different operating frequencies to screen particles or cells of various sizes.

### 4.2. Sorting of Polystyrene Particles with Different Sizes

After verifying the functionality of the SFIT, PDMS with microfluidic channels was bonded to LiNbO_3_ with SFIT, and a sorting experiment on PS particles was conducted using TSAWs generated by the SFIT at different frequencies. In this experiment, three factors—flow rate, voltage, and frequency—affected the final sorting outcome. The effect of frequency on particle sorting was analyzed through the derivation of a formula, where different particles correspond to different driving frequencies. By adjusting the frequency, target particles can be selectively sorted when other conditions are fixed. Voltage affects the strength of a TSAW; the higher the voltage, the greater the amplitude of the TSAW. However, excessive voltage generates heat, which can have two effects. First, in the screening of cells and microorganisms, high temperatures can impact biological activity. Second, LiNbO_3_ is a thermally brittle material, and excessive voltage can cause it to crack during use. An appropriate voltage should be selected based on the sorting voltage operating curve. The effect of flow rate on sorting is reflected in the time (t(TSAW)) that the TSAW acts on the particles. The higher the flow rate, the shorter t(TSAW), and the lower the probability of sorting out the target particles. When the flow rate is too low, impurity particles are also sorted out due to the longer duration of acoustic radiative force. Therefore, it is important to determine the appropriate flow rate for sorting.

**Figure 6:**
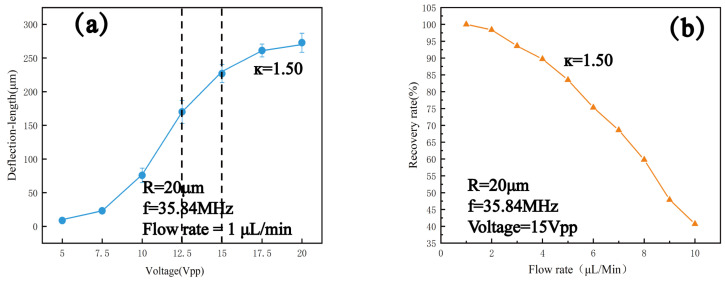
(**a**) Deflection length versus voltage for PS particles with a particle size of 20 μm at 35.84 MHz frequency and a flow rate of 1 μL/min. (**b**) Recovery rate versus flow rate at 35.84 MHz frequency and 15 Vpp voltage for PS particles with a particle size of 20 μm.

At the optimal resonant frequency of 35.84 MHz for the device operation, κ=1.50 for 20 μm microspheres, corresponding to the peak of the acoustic radiative force coefficient. Therefore, 20 μm microspheres were selected to verify the optimal parameters of the operating voltage and flow rate at the operating frequency of 35.84 MHz. We plotted the deflection length as a function of voltage (Figure 6a) and recovery rate (number of target exit particles/number of inlet particles) as a function of flow rate (Figure 6b) using experimental data, and we determined that the optimal voltage for sorting was 15 Vpp and the flow rate was 0.1 μL/min.

**Figure 7:**
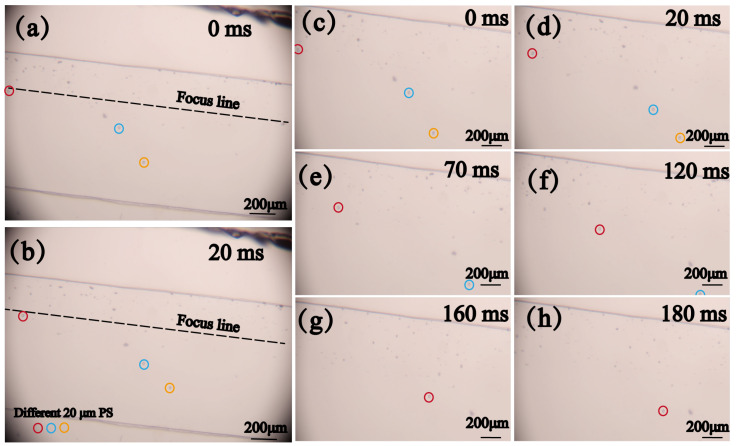
Deflection of PS particles with a particle size of 20 μm at a frequency of 35.84 MHz, a voltage of 15 Vpp, and a flow rate of 1 μL/min: (**a**,**b**) actual images under a 200× optical microscope. (**c**–**h**) enlarged screenshots of the same position at different times.

On the basis of determining the voltage and flow rate parameters, sorting experiments were conducted on mixed microspheres with particle sizes of 5 μm, 10 μm, and 20 μm. A sinusoidal signal with a frequency of 35.84 MHz and a voltage of 15 Vpp was applied to the flowing mixed particles. It was clearly observed that the 20 μm microspheres in the mixed particles were significantly deflected under the influence of the ARF. As shown in Figure 7c–h, the 20 μm PS microspheres were gradually separated from the mixed particles over time and flowed into the target outlet.

**Figure 8:**
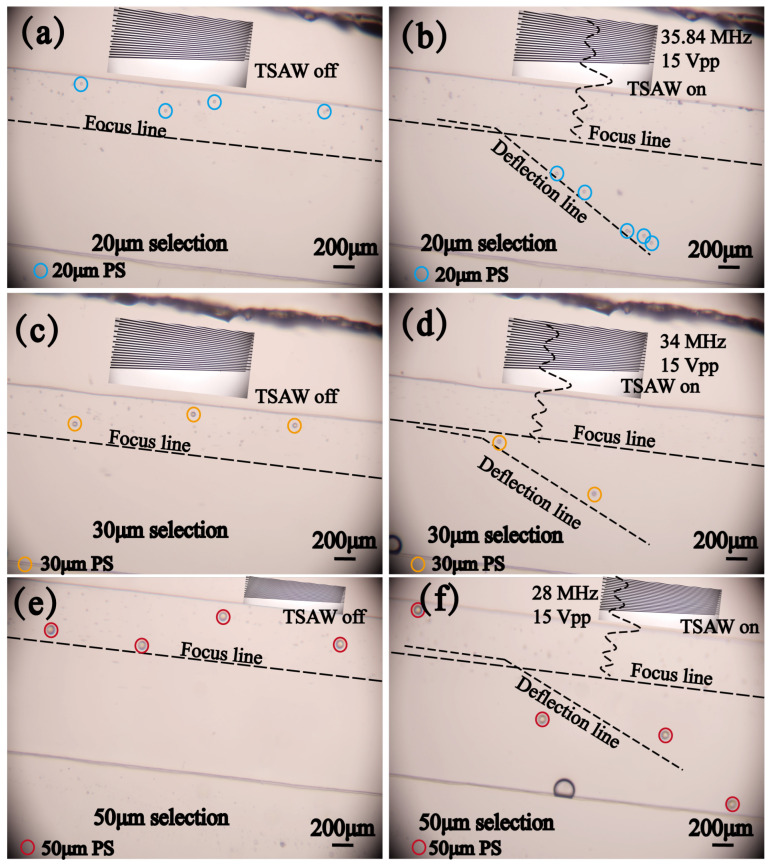
Demonstration of the particle separation process: (**a**) focusing of (5, 10, 20) μm PS mixed particles. (**b**) Separation of 20 μm PS particles. (**c**) Focusing of (5, 10, 30) μm PS mixed particles. (**d**) Separation of 30 μm PS particles. (**e**) Focusing of (5, 10, 50) μm PS mixed particles. (**f**) Separation of 50 μm PS particles.

To verify the broadness of the device’s operating frequency and the purity of the sorting, different frequencies were used to separate 20 μm, 30 μm, and 50 μm PS particles from 5 μm and 10 μm PS particles, respectively. In addition to the parameters used for separating 20 μm PS, a sinusoidal signal with a voltage of 15 Vpp at 34 MHz (κ = 2.14) was used to separate 30 μm PS, and a sinusoidal signal with a voltage of 15 Vpp at 28 MHz (κ = 2.94) was used to separate 50 μm PS. When the TSAW is turned off, as shown in Figure 8a–c, the particles focus on one side of the microfluidic channel under the influence of the sheath flow, creating conditions for the subsequent sorting. When the TSAW is activated, different frequencies are excited at different positions of the SFIT. Based on calculations, TSAW with frequencies of 35.84 MHz, 34 MHz, and 28 MHz can be used to separate 20 μm, 30 μm, and 50 μm PS, respectively. The separation process is shown in Figure 8b–d, where particles of different sizes are deflected at different separation frequencies, allowing them to be separated within the mixed particle flow. The specific separation process can be observed in the recorded video, where 20 μm PS (Video S2), 30 μm PS (Video S3), and 50 μm PS (Video S4) are separated under different TSAWs. This verifies that the designed device has the flexibility of an adjustable operating frequency, and the wide acoustic path makes different operating frequencies more effective, enabling the sorting of particles or cells of varying sizes by adjusting the frequency.

**Figure 9:**
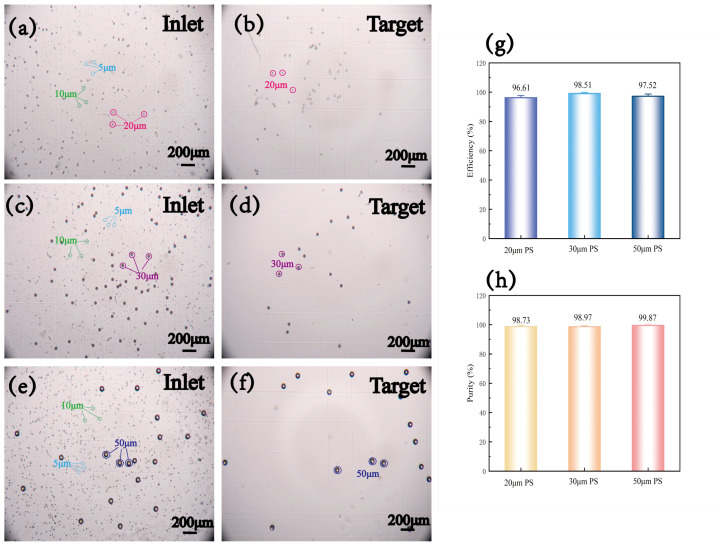
Demonstration of separation results: (**a**) (5, 10, 20) μm PS mixed particles before separation. (**b**) Target exit of (5, 10, 20) μm PS mixed particles after separation. (**c**) (5, 10, 30) μm PS mixed particles before separation. (**d**) Target exit of (5, 10, 30) μm PS mixed particles after separation. (**e**) (5, 10, 50) μm PS mixed particles before separation. (**f**) Target exit of (5, 10, 50) μm PS mixed particles after separation. (**g**) Separation efficiency statistics. (**h**) Separation purity statistics.

By collecting the solutions containing particles before and after separation and observing them on a cell counting plate, we obtained the results shown in Figure 9. After performing statistical analysis on the data from multiple experiments, we calculated the sorting efficiency (number of target exit target particles/number of inlet target particles) and purity (number of target exit target particles/total number of particles at the target exit) of the device. The device achieved a separation efficiency of 96.61% and a purity of 98.73% for 20 μm PS, a separation efficiency of 98.51% and a purity of 98.97% for 30 μm PS, and a separation efficiency of 97.52% and a purity of 99.87% for 50 μm PS. This demonstrates that the device design successfully achieves the goal of sorting particles of various medium sizes.

**Figure 10:**
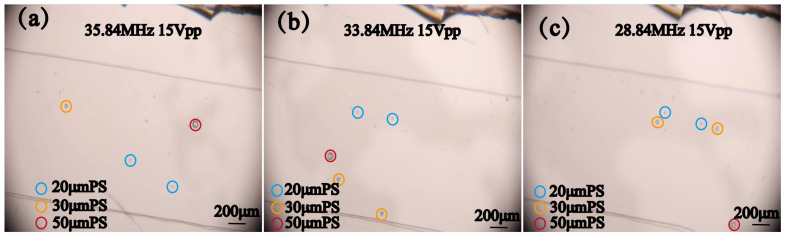
Sorting process of mixed particles with different particle sizes at different frequencies. (**a**) Sorting of 20 μm PS particles at 35.84 MHz frequency and 15 Vpp voltage. (**b**) Sorting of 30 μm PS particles at 33.84 MHz frequency and 15 Vpp voltage. (**c**) Sorting of 50 μm PS particles at 28.84 MHz frequency and 15 Vpp voltage.

In order to verify the possibility of sorting multiple particles in a complex environment, 20 μm, 30 μm, and 50 μm PS particles were mixed together, and 5 μm and 10 μm PS particles were added to make the sorting environment more complex. By adjusting different frequencies, the 20 μm, 30 μm, and 50 μm PS particles were separated from the mixed particle flow during the movement process and sorted out, as shown in Figure 10. The corresponding characteristic frequencies can sort the corresponding particles. However, the simultaneous sorting of all three particle sizes seldom occurs, which, on the one hand, requires more precise calculations and more accurate instruments, and, on the other hand, is also related to the error of manually fabricated devices. Overall, the experiments demonstrated the feasibility of the designed device, specifically its ability to generate a wide acoustic path and cover a large working frequency range. This enables the device to be used in practical applications where particles or cells are of varying sizes, allowing for the operating frequency to be adjusted according to the size of the target particles, thereby enabling the sorting of the target particles.

## 5. Conclusions

In this experiment, a broadband slanted-finger interdigitated transducer (SFIT) with a wide acoustic path and multiple operating frequencies was designed. The SFIT was fabricated on a lithium niobate substrate using photolithographic mask magnetron sputtering and stripping and bonded with a PDMS microfluidic chip designed for unidirectional focusing and multi-channel shunting, resulting in a flexible and tunable sorting device capable of sorting particles, cells, or microorganisms of various sizes. The optimal operating frequency of the device was determined to be in the range of 32–42 MHz, as measured by a vector network analyzer. The device’s performance was verified to exhibit a wide acoustic path and adjustable frequency characteristics within this frequency range. The device was then used to sort 20 μm, 30 μm, and 50 μm PS particles mixed with 5 μm and 10 μm PS, achieving sorting efficiencies and purities above 96%, and verifying the feasibility of sorting one or more components from a complex solution of mixed particles. This provides a solution for practical applications where the size of the particles or cells to be sorted is heterogeneous by efficiently varying the operating frequency to enable sorting. Overall, this work complements the application of slanted-finger interdigitated transducer in microfluidics for sorting across a wide range of particle sizes, and in the future, the sorting range can be adjusted by altering the device’s size, enabling precise device fabrication tailored to specific needs.

## Figures and Tables

**Table 1:** The κ values of PS microspheres with different diameters at different frequencies.

Freq (MHz)	κ				
	5 μm	10 μm	20 μm	30 μm	50 μm
32	0.34	0.67	1.34	2.02	3.36
34	0.36	0.71	1.43	2.14	3.57
36	0.38	0.76	1.51	2.27	3.78

## Data Availability

The original contributions presented in this study are included in the article. Further inquiries can be directed to the corresponding author.

## Supplementary Materials

The following supporting information can be downloaded at https://www.mdpi.com/article/10.3390/mi16040483/s1, Video S1: Experimental results of TSAW path change. Video S2: 20 μm microsphere separation. Video S3: 30 μm microsphere separation. Video S4: 50 μm microsphere separation.
